# Demand Forecasting Approaches for New Contraceptive Technologies: A Landscape Review and Recommendations for Alignment

**DOI:** 10.9745/GHSP-D-22-00334

**Published:** 2023-02-28

**Authors:** Elizabeth LaCroix, Ashley Jackson, Seth McGovern, Kate H. Rademacher, Claire W. Rothschild

**Affiliations:** a Population Services International, Washington, DC, USA.; b PATH, Seattle, WA, USA.; c FHI 360, Durham, NC, USA.

## Abstract

**Introduction:**

Market size estimations and demand forecasts use a variety of methodological approaches to inform decision-making around new (and lesser-used) contraceptive methods. For contraceptive products already available at scale in a market, historical procurement and consumption data can help to inform these forecasts. However, little published guidance is available on appropriate approaches to estimating contraceptive demand in the absence of historical data.

**Methods:**

This landscape review aimed to describe the variety of approaches for modeling demand for new contraceptive methods, highlight opportunities for alignment around forecasting practices, and make recommendations to support more accurate forecasting and sound decision-making based on forecasts. We used the published scientific and gray literature to inform the development of a semistructured guide for key informant interviews. We conducted interviews with 29 experts representing a spectrum of interests in market size estimation and demand forecasting for new contraceptive methods (e.g., ministries of health, donors, manufacturers, technical assistance providers, and demand forecasting specialists). We coded notes from the interviews using thematic content analysis.

**Results:**

The purposes of market size estimation and demand forecasting for new contraceptive methods vary widely, as do associated model inputs and outputs. Key informants revealed a need for more standardized language around market size estimation and demand forecasting and highlighted key recommendations: select models that are fit-for-purpose, clearly articulate assumptions and uncertainty in model outputs, consider a broad range of contraceptive options in a forecast to capture the complete contraceptive supply environment, and perform a reality check of results and refresh assumptions.

**Conclusion:**

We recommend following a simple decision pathway to ensure that forecasts are fit-for-purpose, with appropriate inputs, outputs, and assumptions clearly articulated. Common pitfalls around overestimating demand should be avoided. Incorporating best practices into forecasting exercises will ensure that models are useful for the stakeholders.

## Abstract (precis)

We describe the variety of approaches for modeling demand for new contraceptive methods, highlight opportunities for alignment around forecasting practices, and make recommendations to support more accurate forecasting and sound decision-making based on forecasts.

## Teaser

**Key Findings:**

- The purposes of and methodologies for market size estimation and demand forecasting for new contraceptive methods vary widely. No single approach to forecasting fulfills all purposes, and a mismatch between purpose and methodology can lead to poor decisions.
- Several approaches can help forecasters account for the existing method mix. Some forecasts estimate shifts in the entire method mix and modern contraceptive prevalence, while others generate estimates of uptake or product use for a single method or product.
- Historical trend data from similar contraceptive methods can help ground forecasts for new methods.

**Key Implications:**

- When developing a forecast or market size estimate, it is critical to choose a methodology fit-for-purpose, reality test the results, consider method switching and effects on the broader method mix, communicate assumptions clearly, acknowledge uncertainty, and refresh forecasts as new information becomes available.
- When using a forecast or market size estimate to make decisions, it is essential to confirm if the estimate was developed for this purpose, examine the assumptions in detail, and consider whether adjustments or a different approach are needed.

## INTRODUCTION

In low- and middle-income countries, an estimated 218 million women do not want to have a child in the next 2 years or at all and are not using a modern method of contraception.^1^ When asked why they do not use modern family planning, the 2 most common answers these women give are: (1) health concerns, including health risks, fears of side effects, and unwanted menstrual changes; and (2) infrequent sex.^2^ The introduction of new contraceptive methods has the potential to fill a need for those who would choose a modern method if it better matched their contraceptive preferences (e.g., in terms of side effect profile or on-demand rather than continuous use).

In 2013, Ross and Stover published a landmark analysis showing that across 113 countries, overall national modern contraceptive use increases substantially with the introduction of a new contraceptive method made available to at least half of the country’s population.^3^ The correlation between method introduction and increased contraceptive use should be interpreted with caution because confounding factors may play a role and the added value of each additional method may diminish as options expand. Yet even if the introduction of a new method does not lead to a measurable increase in a country’s modern contraceptive prevalence, it may add meaningful value from both client and health systems perspectives. Some new or modified contraceptive technologies offer higher effectiveness, leading to fewer unintended pregnancies. Some are designed to offer more tolerable side effects or easier use from the clients’ perspective, both of which may improve clients’ quality of life and sexual and reproductive well-being and contribute to more consistent use and, thus, fewer method failures. In addition, some new methods have the potential to address health system failures, such as shortages of trained health workers (e.g., by enabling clients to opt for contraceptive self-administration or administration by lay health workers).

Market size estimates typically provide the number of potential users of a product, and demand forecasts typically provide the number of products needed in a certain time period.^4^^–^^7^ Estimates of market size and demand forecasts are commonly used to inform major decisions related to introducing new (and lesser-used) contraceptive products, from the stages of early research and development investments to the development of plans for initial procurement and launch in country.^8^ Predicting uptake of new contraceptive methods is difficult, as demonstrated by an analysis of U.S. Agency for International Development procurement data showing that demand forecast errors for new and “underused” methods were more than 53 percentage points higher than other methods.^4^ For contraceptive products already at scale in a market, historical procurement and consumption data can inform forecasts. However, little published guidance is available on appropriate approaches to estimating contraceptive demand or future consumption in the absence of such historical data. More accurate forecasting for new methods would help inform decision-making (e.g., at the product development phase) or reduce over- or undersupply, optimizing the use of limited resources.

More accurate forecasting for new contraceptive methods would help inform decision-making or reduce over- or undersupply, optimizing the use of limited resources.

We conducted a landscape review to describe the variety of purposes for and predominant approaches to modeling demand for new contraceptive methods to support sound, forecast-based decision-making and accurate data interpretation. This review builds upon a guide published by the Reproductive Health Supplies Coalition (RHSC) in 2012,^4^ updating recommendations based on the past decade of global experience that includes recent experiences relating to the broader introduction of contraceptive implants,^9^^,^^10^ subcutaneous injectable contraceptives, and the hormonal intrauterine device.^11^^,^^12^ While the RHSC guide focused on demand forecasting to inform immediate procurement decisions, the current review also examines market estimation approaches for contraceptive technologies still in development. By taking a broad look across approaches used to predict demand for new contraceptive methods, we aimed to clarify definitions and spotlight expert recommendations to improve the generation, interpretation, and use of such forecasts from product discovery through introduction.

## METHODS

### Key Informant Sampling and Recruitment

We identified experts in demand forecasting and market estimation for contraceptive products, with a focus on specific expertise in contraceptive products not yet available at scale. Through a consultative process within the study team, we identified an initial list representing expert organizations and individuals. The goal was to maximize diversity of informants across 2 dimensions: (1) to include viewpoints from both “producers” and “consumers” of forecasts to gain varying perspectives on the purpose and process of forecast development and use, and (2) to include informants with varying foci across the contraceptive development pipeline, from very early product research and development to later-stage planning and logistics for product launch in specific markets.

Excluding 1 interviewee who did not respond to our interview request, all other invitees participated. Additional informants were added on an ad hoc basis if recommendations arose during the interviews. We conducted a total of 19 interviews with 29 unique key informants (KIs): 11 interviews with individual KIs, 1 interview with an individual KI via email, 5 interviews with 2 KIs in each interview who had similar roles within the same organization, 1 interview with 2 KIs together representing stakeholders of different types (1 representing a technical assistance provider and 1 a private donor, both of whom were collaborating on a shared forecasting project), and 1 interview with 5 KIs together representing 2 organizations of the same type (both institutional buyers). Three KIs were based in low- and middle-income countries, while the rest were based in high-income countries. Our final sample included KIs representing donors (2 different private donor organizations and 2 governmental), academics (1), technical assistance providers (16), institutional buyers (4 KIs representing 2 different organizations), contraceptive manufacturers (3 KIs representing 2 manufacturers), and 1 Ministry of Health representative.

### Data Collection Procedures

All interviews were conducted via video calls on Microsoft Teams and attended by at least 2 study team members, except for 1 interview in French attended by 1 research team member and another Population Services International staff member fluent in French. Interviews were facilitated by 1 study team member, with another study team member (or, in some cases, multiple) taking detailed written notes during the interview. Written notes were reviewed and expanded upon as needed after the interview, then shared with the full study team for additional comment. In some cases, notes taken by multiple study team members were consolidated.

Interviews followed a semistructured interview guide, which took the form of a slide presentation shared during the interview. The slide deck was initially developed based on the structure of the RHSC guide and updated with more recent peer-reviewed and gray literature identified by the authors.^4^^–^^7^^,^^13^^–^^17^ This was supplemented by the study team’s professional experience. In addition to a list of questions with structured prompts, the interview guide presented informants with draft findings on the following topics: purposes for market sizing and forecasting; types of inputs (i.e., data sources and assumptions) and outputs of market sizing; and forecasting activities (the Supplement includes the slide deck). During each interview, the facilitator described current findings and asked the KI to provide any clarifications, revisions, or additional comments. The deck was refined iteratively, with feedback from each interview incorporated into a new draft, if necessary, with the goal of reaching consensus among our KI experts. We developed additional slides based on KI recommendations for best practices in selecting proxy/analogue contraceptive products and top recommendations for conducting high-quality market sizing and forecasting in subsequent versions of the deck. In total, we developed 4 versions of the slide deck (in addition to a version translated into French). Following procedures used previously in similar landscaping, we chose not to record interviews to encourage participants to share openly, including common challenges and pitfalls of current forecasting approaches and uses—a key aspect of our landscaping review.^18^

We conducted a qualitative analysis of interview notes using thematic content analysis. An initial codebook was developed based on the semistructured interview guide. Two members of the study team coded a subset of notes and then reviewed them jointly to reconcile differences in code application, refine code definitions, and add several additional codes to the codebook. Qualitative analysis was conducted using Dedoose software. The draft manuscript was shared with KIs to give them an opportunity to provide additional comments.

### Ethical Approval

This activity was determined by the Research Ethics Board of Population Services International to not be human subjects research; as a result, no ethical review was required. All KIs were given the opportunity to review the final manuscript and to indicate whether they would like to be acknowledged by name.

## RESULTS

We summarize key themes that emerged from the KI interviews about the considerations, including both challenges and opportunities, associated with demand forecasting for contraceptives.

### Lack of Standard Terminology

The global family planning community has not yet aligned on definitions related to market sizing and demand forecasting. Modeling exercises to estimate procurement needs for supply planning is typically called “demand forecasting”^5^^,^^15^^,^^16^ or “demand estimation.”^14^ Modeling exercises to estimate the potential number of users of a method at a point in the future, or the number of people who would use a method if they had the opportunity today, is often called “market size estimation”^17^^,^^19^ but also called “demand forecasting” by some.

### Considerations for Forecasting Purposes

**FIGURE 1:**
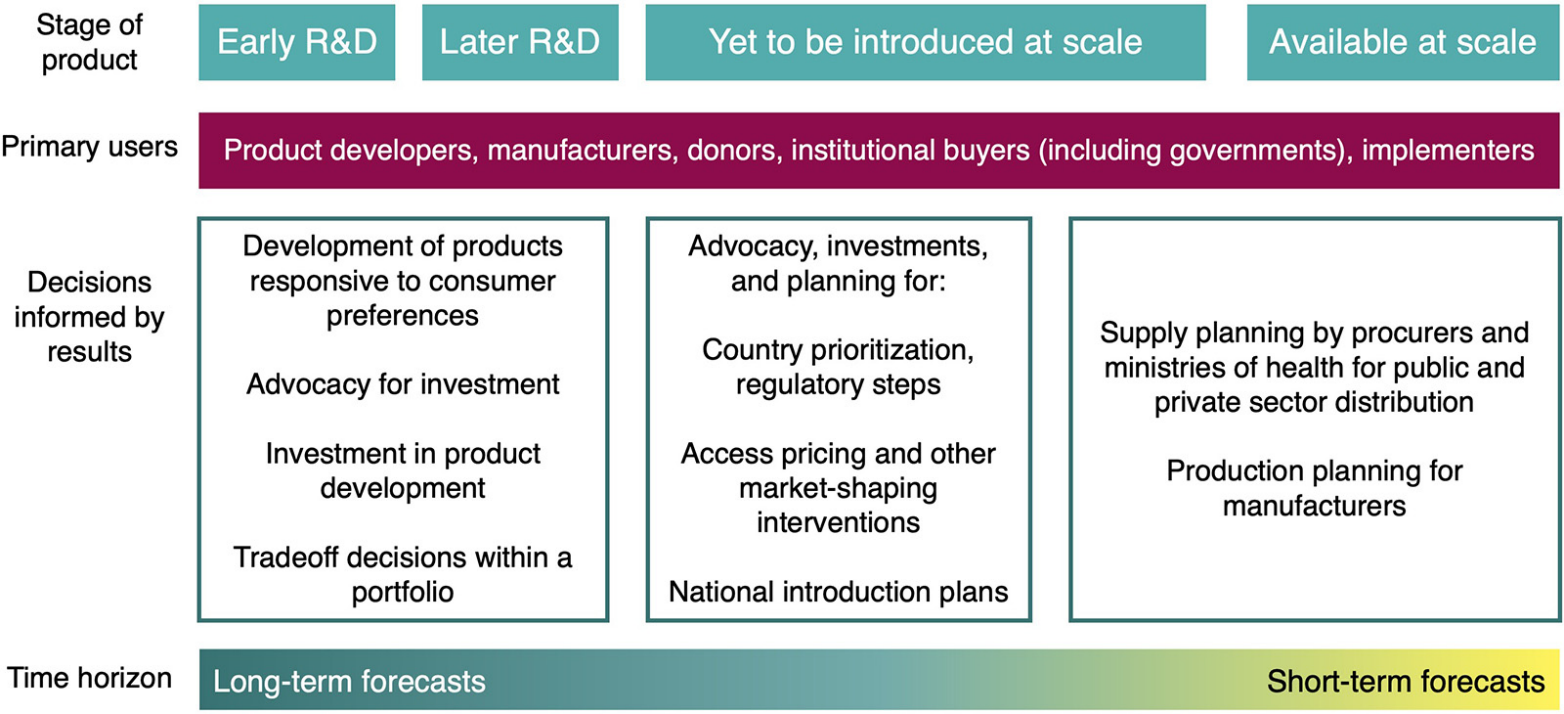
Market Sizing and Forecasting Purposes (Nonexhaustive) Abbreviation: R&D, research and development.

The purposes of forecasting vary across the stages of product development and introduction (Figure 1). KIs stressed the importance of articulating a forecast’s purpose before creating the forecast to ensure actionable and valid results, as the purpose of the model will determine the required inputs, outputs, and assumptions. Across the stages of product development, a variety of primary users will use forecast results to inform a broad range of decisions, including decisions around investment in product development, access pricing negotiations, and national introduction planning (Table). While supply and production planning forecasts are typically associated with existing products in a market, they will inform planning for the scale-up of new products as well. We presented draft versions of Figure 1 and the Table to KIs and incorporated their commentary and feedback into the final versions.

KIs stressed the importance of articulating a forecast’s purpose before creating the forecast to ensure actionable and valid results.

**TABLE:** Illustrative Examples of the Variety of Market Sizing and Forecasting Purposes and Approaches (Nonexhaustive)

**Primary Purpose(s)**	**Inputs, Methods, Data Sources**	**Key Assumptions**	**Outputs**
Investments in research and development (e.g., funders estimating future market size to decide which product development investments to make)	Consumer research (e.g., simulated test market, discrete choice experiment) and research with other stakeholders, overlaid with data on demographic trends	All of the methods under consideration would be widely available in the future.Some assume no discontinuation, and others assume discontinuation rates for analogue products.	Point estimates of number of users by method in a number of countries far in the future (e.g., 20 years out), with 95% confidence intervals
Global market shaping over the near to medium term (e.g., suppliers and donors estimating procurement across priority countries to inform access pricing negotiations)	Bottom-up forecast using historical procurement data for other methods, applying results of market research or pilot introductions to predict uptake relative to these other methods	Procurement of the new method would scale up at comparable rates to findings from market research, pilots, or previous product introductions of other comparable methods.Future growth assumptions account for the timing and scope of product introduction.	Number of units of the product needed over a certain time period (e.g., 5 years) for conservative, moderate, and ambitious adoption scenarios
National introduction planning (e.g., ministry of health developing a costed implementation plan for method introduction)	Separate projections based on:• Service capacity• Consumption of similar product or method • National survey data on current use, unmet need, intention to use, etc.	May assume linear growth, providers would be willing to offer the method, clients with demand would access the method	Multiple linear projections to be reconciled using expert judgment

### Considerations for Model Inputs

**FIGURE 2:**
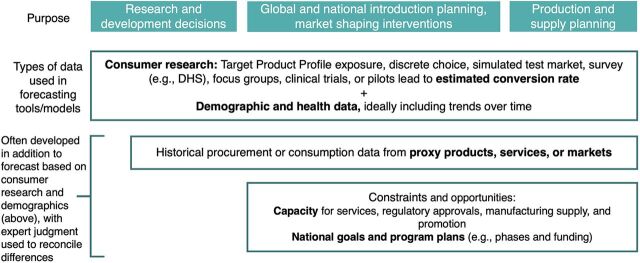
Types of Model Inputs, by Purpose (Nonexhaustive) Abbreviation: DHS, Demographic and Health Survey.

Forecasting models require a variety of different inputs, depending on the model’s purpose and approach (Figure 2). We presented an initial version of Figure 2 to KIs and incorporated their commentary and feedback into the final version. Generally, these types of data include demographic and health data, primary consumer research, historical data, and constraints and opportunities (which look at service delivery capacity, promotion planning/phased introductions, regulatory processes, and national policy). While consumer research, demographic and health data, and historical data tend to be used in models across the product lifecycle, data on constraints and opportunities and national goals tend to be used more in introduction, production, and supply planning.

#### Ambiguity in Using Consumer Research Data

Investments in high-quality consumer research are paramount to inform many types of market size estimates and demand forecasts. KIs noted that while it is needed across all forecasting purposes, consumer research plays a primary role early in the product lifecycle (i.e., during research and development and to inform “go/no-go” decision-making). Consumer research provides data on desired attributes in a contraceptive product, willingness to pay, and, most importantly, likelihood of method uptake. KIs cited numerous methods of consumer research, including discrete choice experiments, simulated test markets, blinded target product profile studies, focus groups, and in-depth interviews. However, there is always a high level of ambiguity with hypothetical user data for methods that have not yet been introduced at scale in real-world settings. Limitations on time and funding to conduct research may also affect the quality of available data.

#### Limitations on Using Demographic and Health Data

Demographic and health data are key to estimating the potential scale of the market and can be used on their own or in conjunction with consumer research. KIs cited censuses and Demographic and Health Surveys as primary data sources; however, they are limited to questions regarding current and past practices. Typical indicators from Demographic and Health Surveys include current method use and unmet need for contraception but rarely include new contraceptive methods. KIs noted that demographic data from censuses could be combined with consumer research regarding uptake to estimate the market size/demand for a new contraceptive product.

#### Lack of Alignment on Historical Procurement and Consumption Data

Historical procurement and consumption data are preferred for forecasting products already available in a market. Several KIs stated the importance of distinguishing between procurement and consumption data, noting that estimates from procurement and consumption do not necessarily align. Consumption happens on a longer time scale than procurement—there may be months or years between product procurement and consumption. To reconcile differences between historical and consumption-based forecasts, many KIs recommended triangulating with additional data and bringing in expert opinion.

To reconcile differences between historical and consumption-based forecasts, many KIs recommended triangulating with additional data and bringing in expert opinion.

When a contraceptive method enters a new market where historical data for this market do not exist, data from other countries can be used to help estimate uptake trends and set price points. For entirely new contraceptive methods, historical data on a proxy or analogue product can be used, such as looking at intramuscular injectables to estimate the market for subcutaneous injectables. KIs noted the importance of choosing an appropriate proxy product, recommending selecting a product that is similar to the new product in terms of value proposition, channels of access, price point, and frequency of use (Box 1). An initial version of Box 1 was developed after KIs offered recommendations for choosing proxy products; the initial version was incorporated into subsequent interviews to elicit additional feedback.

BOX 1 **Recommendations for Selecting Proxy Products**

**Select proxy products that are similar to the new product in terms of:**

- Value proposition
- Channels of access
- Price
- Frequency of use

**Pilot studies and consumer research can inform selection of proxy product:**

- Which methods did early adopters switch from?
- Which methods are current users most interested in?

**Look at how uptake of proxy product changed over time:**

- Uptake of new methods tends to follow an S-curve with slow initial uptake, then a surge in growth, then a plateau
- Do not expect a new product to quickly reach volumes similar to those of a long-established product

#### Other Constraints and Opportunities

KIs agreed on the importance of considering contextual factors, such as capacity for service delivery, regulatory approvals, promotion plans, policy decisions, funding environment, and product attributes, in making realistic forecasts. Capacity for service delivery at the facility level (e.g., number of providers trained to offer a method) can act as another triangulation point or a reality check for a consumption or procurement-based forecast. KIs suggested comparing discrepancies between implementation capacity and the volume of product requested. Regulatory status, product promotion plans, and national policy decisions can affect the rate of growth of adoption positively or negatively within the constraints of the current funding environment. Attributes specific to the contraceptive method may also affect uptake, such as cold chain requirements or ancillary products required (e.g., pregnancy tests and equipment for insertions or removals).

#### Application of Discounting Approaches to Avoid Overestimation

Many KIs noted the importance of applying discounting approaches to not overestimate the potential market size. For procurement-based data, it is important to consider supply chain wastage, while for consumption data, discontinuation should be taken into account. Consumer-based research may correct for overstatement, including discounting the likelihood of uptake with factors like satisfaction with their current existing method. Discounting assumptions around access and product awareness can account for the fact that access and awareness will be low at the beginning of a product introduction and grow over time as the product reach expands.

### Considerations for Model Outputs

#### Selection of Units and Conversions Between Output Units

Market size estimations and demand forecasts produce estimates in a variety of output “units.” Common output units identified by KIs can be broadly grouped into 3 categories: number of commodities, market share, and return on investment. Model outputs are often determined by stage in the product lifecycle and the intended consumer of the model. Several KIs noted that the output units of the model would typically vary across the product lifecycle. Models may also produce several different output units used in combination for decision-making.

Market sizing to quantify the total addressable market is commonly used in early-stage product development. For example, donors and innovators may use market size estimations to rank the promise of products early in the research and development lifecycle to make investment decisions and portfolio tradeoffs. The goal of such market sizing is to understand a new method’s value proposition, including target segmentations, total numbers and patterns of potential growth of users over time, and peak potential market share. KIs from the technical assistance and manufacturing sectors also highlighted return on investments in terms of costs and profits as key model outputs for early-stage products. KIs from 1 manufacturer confirmed that models were often used to assess return on investment as the single most important consideration. Such models may estimate disaggregated costs and profits to specify return on investment to different actors. Total profits, gross versus net sales, and investment “break-even” points under different scenarios may also be valued outputs, particularly for investors and manufacturers. At later stages in the product lifecycle, including supply planning, procurement, and product launch phases, emphasis shifts from market share and number of product users to number of product units.

Many KIs discussed processes by which output units may be converted to other units of interest. The most common conversion described was from estimated number of users to number of commodities (or vice versa). Several KIs described this conversion as the greatest challenge and largest miss for market sizing and demand forecasts. A common approach to this conversion is to apply the couple-years of protection (CYP) conversion factors, which estimate method-specific protection from pregnancy over a 1-year time frame.^20^^–^^22^ Method-specific discontinuation rates may also be used.

The use of CYPs requires 2 major assumptions, identified as challenges by KIs. First, depending on the stage of product development, new products may not have empirically based CYPs. In such cases, the CYP of an analogue product may be used. Second, these factors are standardized across global geographies; as such, they may not accurately represent context-specific trends in method discontinuation or adherence. CYPs may also fail to accurately capture minor lapses in use—for example, an individual who reports continuous use of a method may fail to report minor instances of lack of adherence (i.e., missed pills or delays in picking up a resupply of the method); overall, these minor deviations between reported use and actual consumption can compound for short-acting methods that require regular resupply, resulting in a model that substantially overestimates product units required from the number of estimated users. On the other hand, 1 informant representing the technical assistance sector emphasized the need to account for product wastage, which requires adding an additional buffer to ensure available product for all intended users.

Other conversions between unit types were described as straightforward in practice: for example, product units may be converted to revenue using a simple conversion factor based on unit price. However, even this more straightforward conversion requires assumptions. One KI noted that, for products without established prices, data to assess price elasticity of demand for the product—such as through experimental methods like discrete choice experiments—could improve the quality of monetary outputs.

#### Time Horizon of Model Outputs

Model purpose was a key determinant of the time scale for model estimates, with time periods ranging from quarterly (for procurement purposes) to more than 20 years (for donor investment decision-making). Product phase, rather than calendar time, can also be used to produce dynamic estimates for various stages of introduction (e.g., pilot vs. national). One KI from the technical assistance sector argued that providing dynamic estimates over time is a requirement of all true forecasts, while others reported that single point estimates are used for simplicity.

#### Lack of Standard Approach to Convey Model Uncertainty

While nearly all KIs agreed that clearly conveying uncertainty in model estimates is important, there was a lack of standard approaches for doing so. Prevalent approaches ranged widely in complexity and depended on output type. For models estimating number of users or product units, the most common approach described was to provide a midlevel (average) estimate with “best” and “worse” case scenarios. KIs reported presenting several models with varying assumptions to represent more or less conservative key model assumptions. KIs highlighted that conservatism in model assumptions may differ by model purpose. For example, KIs representing manufacturers cited use of estimates of the minimum sales needed to avoid financial losses as the “worst case scenario,” which is used to estimate the probability of breaking even. In contrast, 1 KI noted that a procurement “buffer” is always added to the procurement estimate to prevent possible stockouts. Several KIs cited more complex approaches to describing uncertainty, including using simulation approaches (e.g., multiple probability/Monte Carlo simulations) to model full point estimate distributions and confidence intervals, noting that such approaches are not widely employed.

While nearly all KIs agreed that clearly conveying uncertainty in model estimates is important, there was a lack of standard approaches for doing so.

### Recommendations for Best Practices

Common themes emerged related to selecting a specific model approach, using best practices in developing and validating the model, and disseminating model results (Box 2). Based on key insights and recommendations from KIs, we developed an initial version of Box 2 that was incorporated into subsequent interviews to generate additional commentary.

BOX 2 **Top Key Informant Recommendations**

**In selecting approach:**

- Define terms to facilitate shared understanding among all stakeholders
- Decide upon and communicate a clear purpose and intended use of model(s)
- Select your approach based on this purpose and resource constraints

**When conducting the forecast or market size estimate:**

- Consider method switching and effects on broader method mix
- Reality test the forecast results (e.g., based on consumer data, analysis of capacity, historical consumption of other products, funding realities)

∘ Especially important when forecast feeds into supply plan

**In communicating and/or using results:**

- Clearly articulate assumptions
- Acknowledge uncertainty
- Refresh the forecast once you know if assumptions were correct

#### Select Modeling Approaches That Are Fit-for-Purpose

All informants agreed that successful models are designed for the specific needs of a particular model consumer (e.g., donor, procurement agency, or government). Many informants recommended working intensively and continuously with the forecast consumer throughout model creation to clarify the purposes of the forecast, identify evolving needs over time that may impact modeling approaches, and clearly understand desired outputs to drive decision-making.

#### Situate Forecasts Within the Full Range of Contraceptive Options

Although several KIs described forecasts to estimate market sizing for the full range of contraceptive methods, KIs reported that most forecasts focus on a single or selected set of methods. Nevertheless, KIs emphasized the importance of making realistic assumptions about method switching—sometimes referred to as “market cannibalization”—to acknowledge that new contraceptive products must be contextualized within the full current and future contraceptive market. Several KIs mentioned that failure to adequately account for and contextualize other available contraceptive methods was a common pitfall of “naïve” forecasts. This contextualization includes appropriate estimation of conversion—or switching—rates from existing methods to a new product and rates of uptake by contraceptive-naïve users as an entire workstream in and of itself; launch experiences for analogue or reference products in similar markets are often used to guide assumptions.

#### Perform a Reality Check of Results and Refresh Model Assumptions

Many KIs emphasized the importance of “reality checking” model results by looking at the capacity of service delivery and implementation planning. Provider and facility capacity can be a rate-limiting factor for growth in adoption for products requiring a prescription or provider insertion. Several KIs noted the importance of implementation planning as well, including number of facilities, capacity for provider training and supportive supervision, funding environment, product distribution channels, and consideration of the marketing plan. In the case that the reality check is different than the original forecast, expert judgment can be used to reconcile the differences and provide context.

Many KIs emphasized the importance of “reality checking” model results by looking at the capacity of service delivery and implementation planning.

Once a new product is introduced, there are more data available that can be used to evaluate and revise forecast estimates. Several KIs described processes for assessing forecast accuracy dynamically—either during a specific stage of product introduction or on a routine (e.g., annual) basis. Several KIs described the use of specific accuracy thresholds: forecasts are revised if early estimates deviate from actual product launch data in the early product introduction phases; forecast assumptions can also be compared to data collected during product launch to assess validity.

#### Clearly Articulate Assumptions

Assumptions are inherent to market sizing and demand forecasting, with increasing number of assumptions leading to higher level of uncertainty. KIs emphasized the need for, as 1 KI termed it, “responsible model hygiene,” in which modelers are up-front about both what assumptions were used and the certainty or level of precision in those assumptions. For this reason, KIs emphasized the importance of conducting sensitivity analyses and emphasizing the level of impact that core assumptions have on model estimates. One KI described that the value of the market sizing and demand forecasting models is nearly entirely in the quality of the methods used to estimate the input parameters and that models should be built with “stopping points” so that consumers can clearly see the sequential impact of each input. Several KIs called for more transparency among organizations that produce and use models to increase transparency and understanding of when and why model estimates diverge and to build “public goods” to improve the quality of forecasts.

## DISCUSSION

The results of this review provide expert recommendations and suggested best practices for forecasting demand for new and lesser-used contraceptive technologies, building upon the RHSC guide from 2012 to include estimation for products still in the early phases of research and development.^1^ We recommend using a consistently applied, shared vocabulary around market sizing and demand forecasting. We lay out a simple decision pathway for how to develop a forecasting approach, draw attention to some key potential pitfalls, and suggest best practices for using and interpreting forecasts.

### Harmonize Definitions

We noticed that across the existing literature, and even among our KIs, there was inconsistent terminology used when discussing market sizing and demand forecasting. Forecasting is often used as an umbrella term to encompass market sizing, demand forecasting, and quantification activities. Makers and consumers of forecasts come from a variety of backgrounds and may not have an academic background in forecasting. We propose consistent use of the following definitions to avoid misinterpretation and misuse of the results of market size estimation and demand forecasting.

- **Market sizing** or **market size estimation** is the process of estimating the number of potential users of a product, typically early in the product lifecycle.^17^^,^^19^
- **Demand forecasting** is the process of estimating the quantities of products that will be needed in a given time frame among a certain population or within a defined geography.^4^^–^^6^
- **Supply planning** is the process of calculating the product quantities required to fill the supply pipeline, scheduling shipments, and estimating costs.^4^^–^^6^^,^^13^
- **Quantification** encompasses both demand forecasting and supply planning to determine the total number of products to procure, taking into account funding availability.^4^^–^^6^^,^^13^
- **Conversion** or **uptake rate** is the estimated percentage of people in a group (such as current users of a given contraceptive method or nonusers of contraception in a particular demographic segment) predicted to switch to using the new method.
- **Proxy** or **analogue products** are contraceptive products that have historical consumption and/or procurement data and share key characteristics with the new product of interest.

### Develop a Fit-for-Purpose Model

**FIGURE 3:**
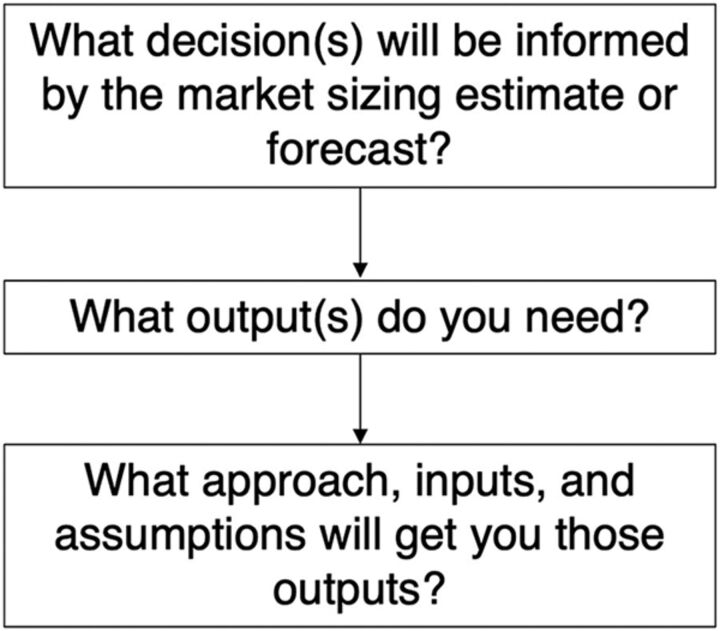
Decision Pathway for Forecast Development

The KIs emphasized the importance of choosing a model that is fit for purpose for the intended forecast consumer, including getting buy-in and understanding among stakeholders. To ensure that important considerations are not overlooked, we recommend implementing a simple decision pathway to develop a market sizing or forecasting approach (Figure 3). Steps in the process include: describing the goal of the forecast, deciding on the necessary outputs, and finally, determining and obtaining stakeholder agreement on the required inputs and assumptions.

When working with stakeholders, determine the question being answered, such as whether to invest in research and development for a new product (Table). One KI noted instances where volume guarantees were negotiated based on consumption-driven forecasts, leading to concerns when countries did not order the planned number of products. Another KI noted an instance where demand exceeded the negotiated volume guarantee, and production has not increased to meet demand. Ultimately, the decision being made is the driver behind the purpose and approach of the forecast. Deciding whether to invest in developing a new product requires a different approach than creating a national introduction plan for a new product.

Once the key decision is clearly stated, determine what type of output(s) will provide the necessary decision data. As several KIs noted, the “unit” of the output often varies across the product lifecycle. It can be difficult to convert effectively between output types, particularly from products to users (and vice versa), and can lead to overestimates of demand and use. To avoid this scenario, we recommend determining the type of output needed from the start and designing the forecast around that determined output type. For example, when conducting market sizing for a research and development investment decision, the output may be a point estimate of number of potential users across multiple countries at a certain point in the future (Table).

We recommend determining the type of output needed from the start and designing the forecast around that determined output type.

It is also important to account for and consider uncertainty around the outputs—consider how a range of uncertainty will affect the forecast decisions. Among the methods KIs presented, the most common and one of the easiest to implement is creating an average estimate with “best” and “worst” case scenarios. When designing these scenarios, it is important to make clear and articulated assumptions about what constitutes the best and worst cases.

After the purpose is determined and the type of output decided upon, the inputs and assumptions can be developed. When investing in new product research and development (Table), using estimated conversion rates is one of the most important types of data to determine the potential market size and consumer demand. In an ideal world, these data would be collected directly from consumers via clearly designed quantitative research, such as discrete choice experiments or simulated test markets. However, resource constraints are often a limiting factor. Data from preexisting surveys (such as Demographic and Health Surveys) can be used to estimate current use and intent to use but do not capture potential conversion rates. In some cases, historical data from a proxy product can be used to estimate demand, but as KIs noted, choosing an appropriate analogue product is not necessarily straightforward and involves its own set of best practices (Box 1).

Assumptions should be based on available data and expert knowledge, clearly articulated, and agreed upon by stakeholders. Changes to assumptions can dramatically affect the forecast results. Altering assumptions is a useful way to discuss uncertainty, providing a range of outputs across scenarios. We recommend clearly documenting assumptions and making them publicly available with the forecast whenever possible.

The original World Health Organization approach to contraceptive introduction included the broader service delivery system as a key variable to constrain or expand opportunities for contraceptive introduction or scale-up.^23^ These contextual factors remain an important consideration today: as KIs noted, it is important to reality test the model and assumptions, accounting for service delivery capacity and taking into consideration funding realities as potential rate-limiting factors. In cases where the reality check is different from the original forecast, expert opinion can be brought in to reconcile the differences. Finally, it is important to update the forecast when new data are available. Once a new product is introduced in a market, previously nonexistent data can replace original assumptions around use and consumption.

It is worth noting that many forecasts are used pragmatically and not designed for the purpose of contributing to generalizable knowledge but to inform decisions in an investment portfolio or to inform decision-making in government. Programmatic documentation of forecasting processes varies in practice and quality. Often this means that market size estimates and forecasts do not get published in peer-reviewed journals but end up in the gray literature, if they are shared at all. In some cases, forecast models may be proprietary and confidential. However, developing shared standards for forecast practice, reporting, and transparency (whenever possible) can allow forecasters to better learn from each other and ultimately build more accurate forecasts.

Developing shared standards for forecast practice, reporting, and transparency can allow forecasters to better learn from each other and ultimately build more accurate forecasts.

### Avoid Common Pitfalls

While following the decision pathway above can lead to clearer and more valid forecasts, there are additional factors that can affect the accuracy of a forecast. KIs noted that to avoid overestimates in market sizing and demand, account for the following: overstatements in consumer research, method discontinuation, and uptake in the context of the entire method mix.

It is important to account for overstatements among respondents to correct for overestimation, exaggeration, and desirability bias when incorporating consumer research data.^24^ One approach noted by a KI is to weight survey responses regarding likelihood of method uptake (e.g., discounting “I would probably use” more heavily than “I would definitely use”); another option is to consider consumer satisfaction with their current method of contraception—those satisfied with their current method would be unlikely to adopt the new method.

Data on product consumption, particularly failure to account for discontinuation, can also contribute to overestimates of demand. For new contraceptive methods, there will be limited to no data on discontinuation rates, and applying discontinuation rates for an appropriate proxy product with a similar refill profile can reduce overestimation errors. However, even small periods of product discontinuation use can scale up to substantial impacts on a forecast. While a consumer may report consistently using a method of contraception, there still may be extra time between refills or skipped doses. At an individual level, this may add up to days or weeks over the course of a year. One KI noted that individuals may easily overestimate their yearly use by an entire month. When applied to a scaled-up forecast, this could lead to a substantial difference in actual use, regardless of the accuracy of other model inputs and assumptions.

Finally, many forecasts only focus on a single method. Study participants who are not aware of the full range of existing contraceptive options may say they would use the new method when they would actually be more interested in adopting an existing method if it were easily accessible and presented in the same way. A single-method demand forecast can also fail to account for the likelihood of “market cannibalization”—users switching from an existing method to the current method, which would reduce the impact of the new method on the modern contraceptive prevalence rate. Where possible, KIs emphasized the importance of not only estimating uptake of the new contraceptive method but contextualizing uptake within the full contraceptive method mix to better understand the likely uptake and the balance of costs and benefits associated with introduction.

### Interpret Existing Forecasts Carefully

Previously developed forecasts can provide valuable information and reference points for discussing a market. However, 1 KI noted that people often view forecasts as “monolithic” rather than individualized and nuanced, contributing toward wanting to use 1 forecast to answer many questions. With the added difficulties in obtaining data and the considerable time and resources required to develop a forecast, it is tempting to repurpose an existing forecast. The importance of using a fit-for-purpose model for decision-making cannot be overstated. We caution against using estimates from existing forecasts without examining their original intent and underlying assumptions and determining if they can be used to answer the current question.

We caution against using estimates from existing forecasts without examining their original intent and underlying assumptions and determining if they can be used to answer the current question.

## CONCLUSIONS

Great strides have been made in market sizing and demand forecasting for new and lesser-used contraceptive methods. In the last decade, we have gleaned lessons from the introduction of contraceptive implants, subcutaneous injectable contraceptives, and hormonal intrauterine device products. Common forecasting issues, such as overestimating demand and applying existing forecast data in a not-fit-for-purpose way, can be addressed to improve decision-making. There are numerous opportunities to apply these practices to new contraceptive products under development, such as long-acting vaginal rings or contraceptive creams or gels^25^ and multipurpose technologies that could provide protection from both sexually transmitted infections and unintended pregnancy.^17^

## Supplementary Material

GHSP-D-22-00334-Supplement.pdf

## Notes

**Peer Reviewed**

**First published online:** February 10, 2023.
