# Supplementary material for: Demand Forecasting Approaches for New Contraceptive Technologies: A Landscape Review and Recommendations for Alignment
Source: Glob Health Sci Pract. 2023 Feb 28;11(1):e2200334. doi: 10.9745/GHSP-D-22-00334 (PMC9972375; doi:10.9745/GHSP-D-22-00334)
Supplement: GHSP-D-22-00334-Supplement.pdf [file GHSP-D-22-00334-Supplement.pdf]

Supplement to: LaCroix E, Jackson A, Seth McGovern, Rademacher KH, Rothschild CW. Demand forecasting approaches for new contraceptive technologies: a landscape review and recommendations for alignment. *Glob Health Sci Pract*. 2023;11(1):e2200334. https://doi.org/10.9745/GHSP-D-22-00334

Market Sizing and Demand Forecasting for New Contraceptive Technologies: Landscape Review

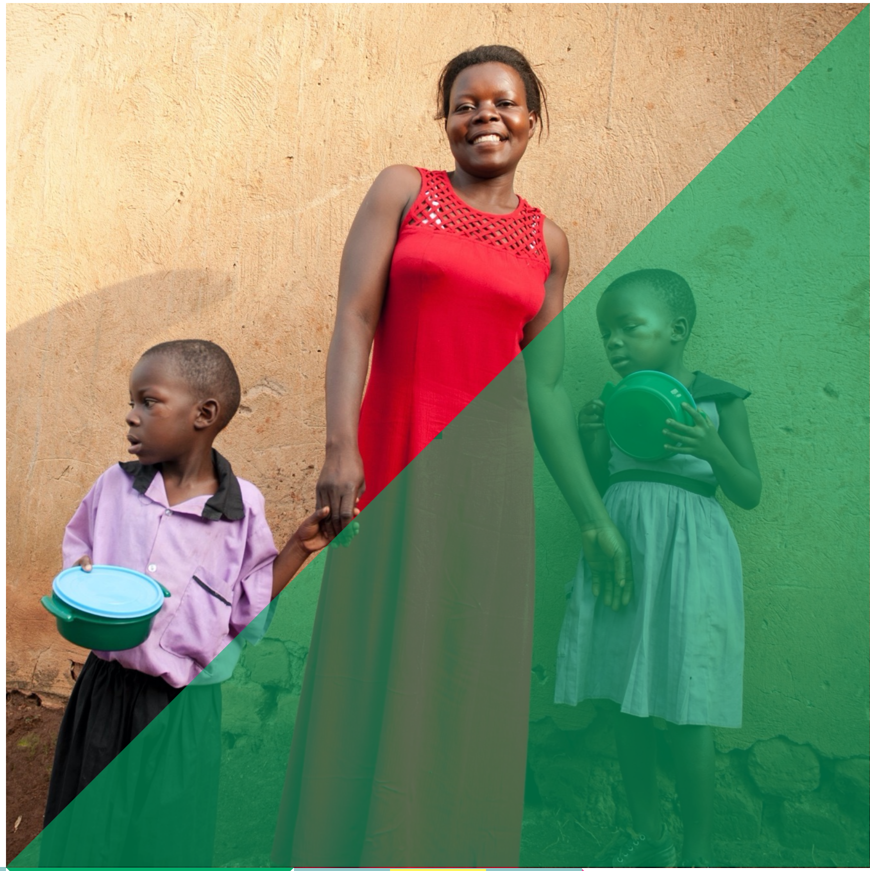

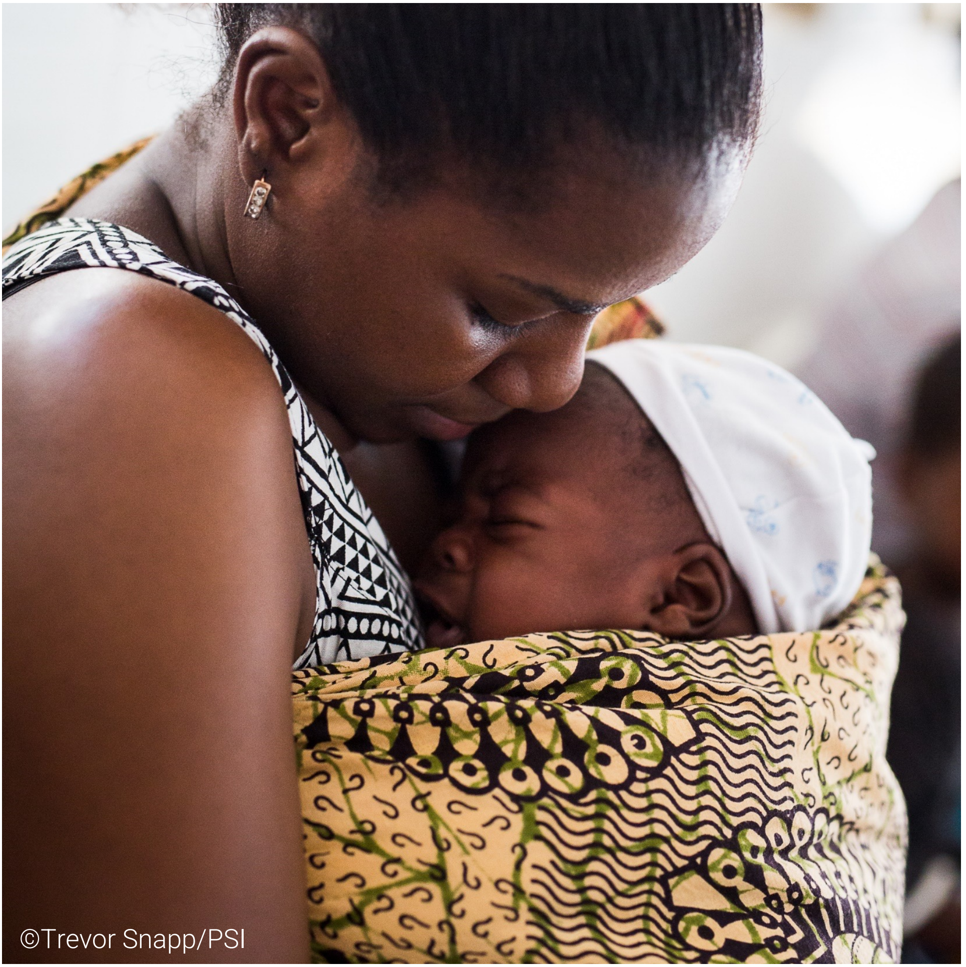

AGENDA

- Objectives
- Process
- Interview questions
- Feedback on initial analysis of the landscape

OBJECTIVES

To strengthen the global health community's understanding and use of market sizing and demand forecasting for new* contraceptive technologies, LEAP and EECO will:

- Examine the methods, assumptions, and purposes of common forecasting approaches
- Describe common forecasting approaches
- Recommend how to choose an approach fit for purpose

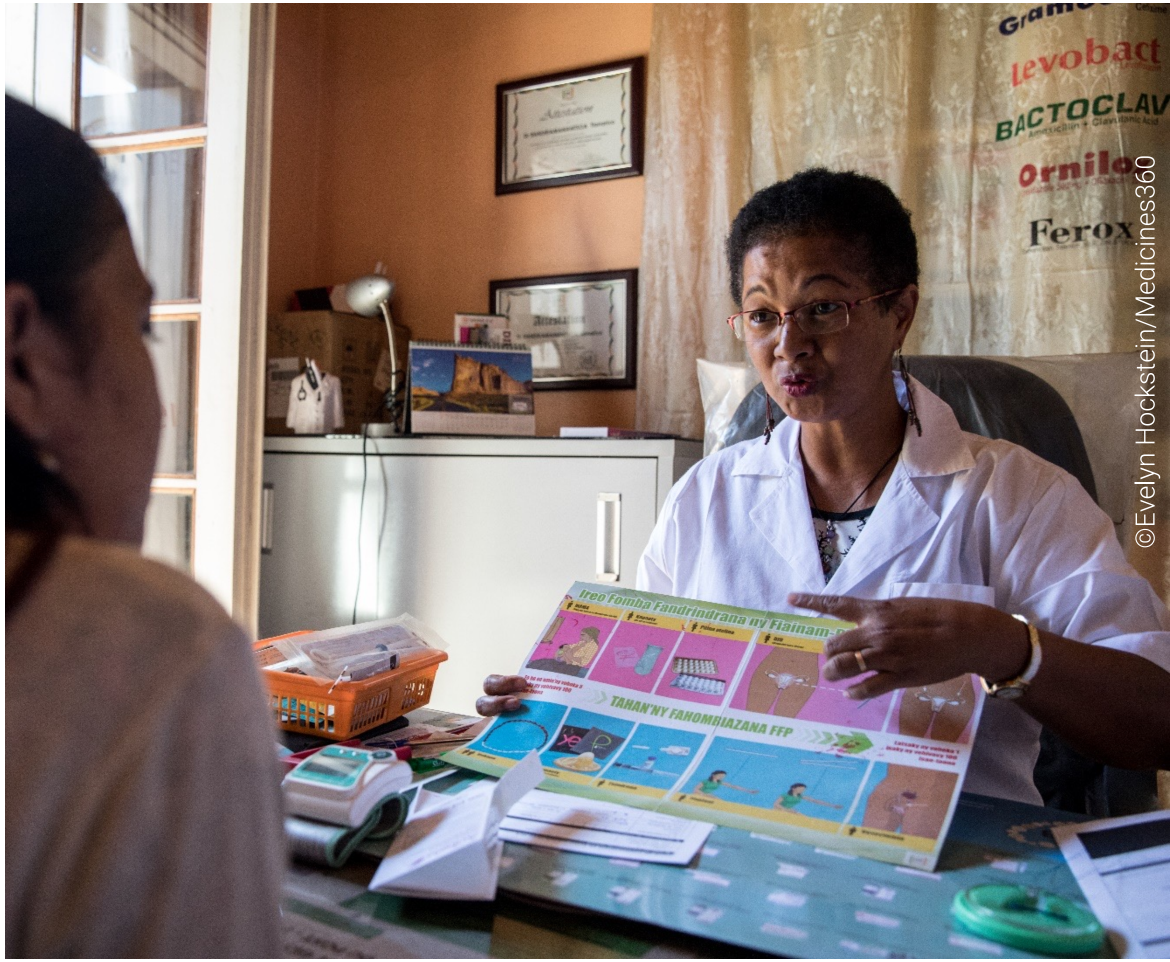

** and older technologies that are not yet at scale*

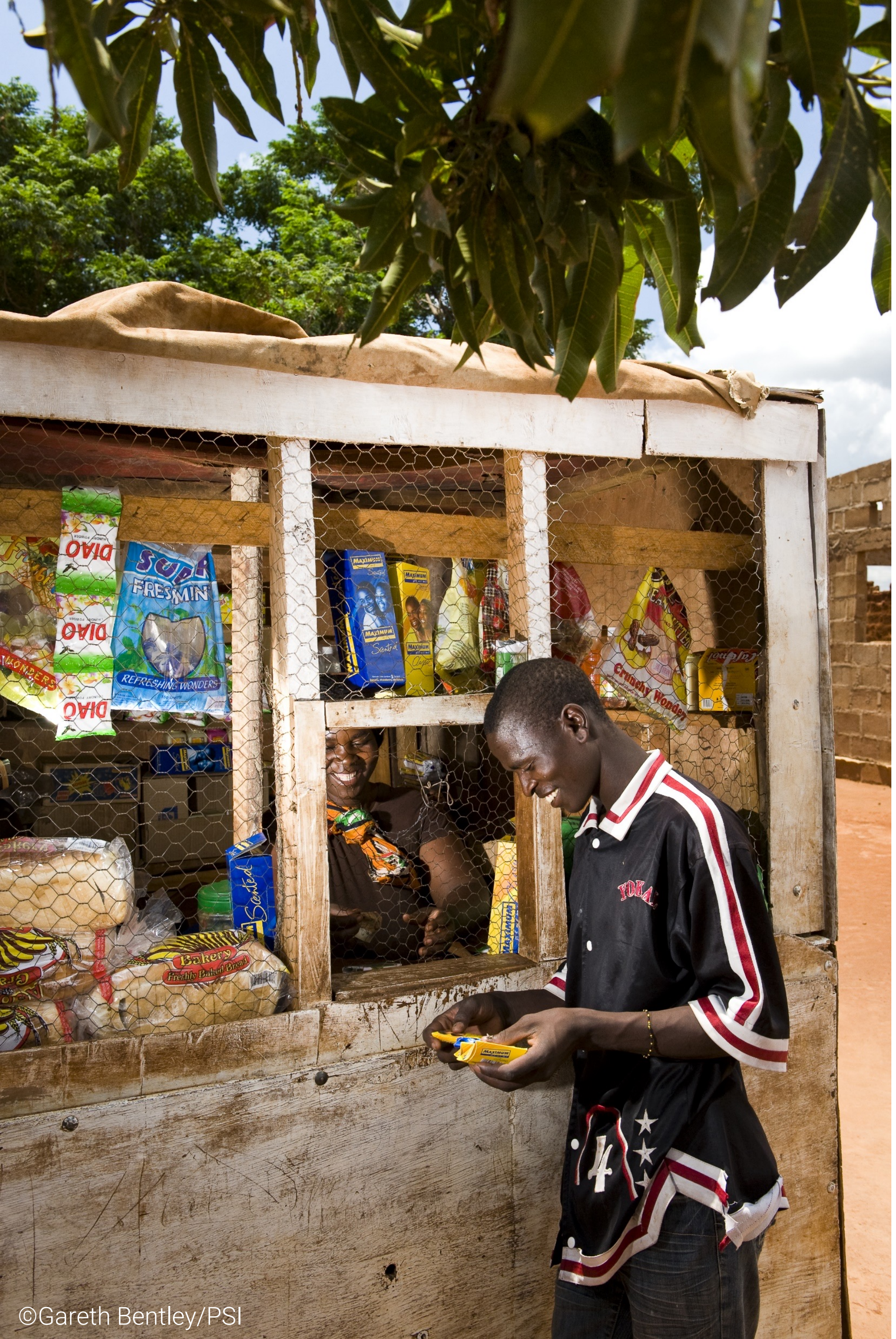

©Gareth Bentley/PSI

PROCESS

1. Landscape review

2. Draft slide deck
   - Clarification of key terms/concepts
   - Conceptual framework for describing and applying models

3. Key informant interviews with experts to refine and iterate upon analysis

4. Development of a written document (e.g., manuscript, guide)

5. Review of final slide deck and document; publication and dissemination

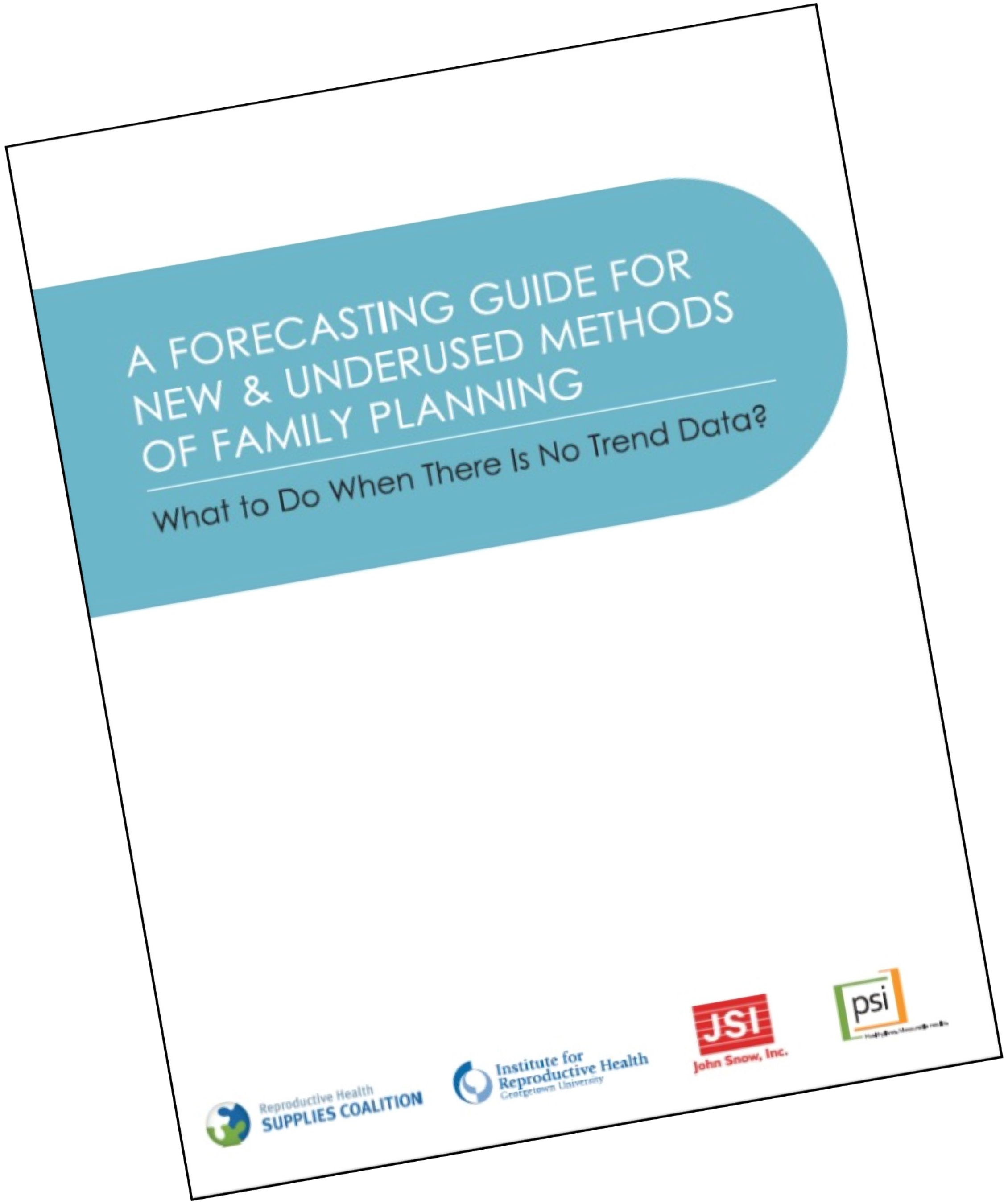

Institute for Reproductive Health, Georgetown University (IRH/GU), John Snow Inc. (JSI), and Population Services International (PSI) for the Reproductive Health Supplies Coalition (RHSC). 2012. *A Forecasting Guide for New & Underused Methods of Family Planning: What to Do When There Is No Trend Data?* Washington, DC: IRH/GU, JSI, and PSI for the RHSC. https://marketbookshelf.com/wp-content/uploads/2017/05/RH-supplies-_-A-Forecasting-Guide-for-New-and-Underused-Methods-1st-Edition.pdf

INTERVIEW QUESTIONS

- What approach(es) have you used to forecast the use of contraceptive products for which we have no historical data?
- What are the key assumptions of this approach?
- Why and when would you recommend selecting this approach over alternatives?
  - In what situations (if any) would you not recommend it?
- What type of data is required and/or optimal for this approach?

DECISION PATHWAY

1. What decision(s) will be informed by the market sizing estimate or forecast?

2. What output(s) do you need?

3. What approach, inputs, and assumptions will get you those outputs?

MARKET SIZING & FORECASTING PURPOSES

Stage of product

- Early R&D
- Later R&D
- Yet to be introduced at scale
- Available at scale

Primary users

Product developers, manufacturers, donors, institutional buyers (including governments), implementers

Decisions informed by results

Development of products responsive to consumer preferences

Advocacy for investment

Investment in product development

Tradeoff decisions within a portfolio

Advocacy, investments, and planning for:

Country prioritization, regulatory steps

Access pricing and other market shaping interventions

National introduction plans

Supply planning by procurers and Ministries of Health for public and private sector distribution

Production planning for manufacturers

Time horizon

Long-term forecasts → Short-term forecasts

OUTPUTS

- **Users** vs. **units procured** vs. **units consumed** vs. **monetary value**
- **One moment in time** vs. **growth over time**
  - Widely varying time horizons (3 months - 20 years)
- **Numerical** vs. **statistical** vs. **ratings**
  - Single point or line estimate
    - With or without confidence interval or distribution
  - Estimates for different scenarios (e.g., ambitious, conservative)
  - Ratings (e.g., green/yellow/red) of how "promising"
  - Relative market share
  - Stochastic simulation (e.g., Monte Carlo)
  - Breakeven point
- **Single method/product** vs. **entire method mix**
- **Disaggregation**
  - Geographic scope, market segments
  - Channel or mode of use (e.g., public, private, self-administered)

With any output type, it is important to acknowledge uncertainty and avoid implying false precision

Forecasts for entire method mix are important for decisions about prioritization of funding

INPUTS

Purpose	R&D decisions	Global and national introduction planning, market shaping interventions	Production & supply planning

Types of data used in forecasting tools/models

Consumer research: Target Product Profile (TPP) exposure, discrete choice, simulated test market, survey (e.g., DHS), focus groups, clinical trials, or pilots lead to **estimated conversion rate**

+

Demographic and health data, ideally including trends over time

Often developed in addition to forecast based on consumer research and demographics (above), with expert judgment used to reconcile differences

Historical consumption data from **proxy products, services, or markets**

Constraints & opportunities:
Capacity for services, regulatory approvals, manufacturing supply, and promotion
National goals & program plans (e.g., phases, funding)

SELECTION OF PROXY/ANALOG PRODUCTS

- Select proxy products that are similar to the new product in terms of:
  - Value proposition
  - Channels of access
  - Price
  - Frequency of use
- Pilot studies and consumer research can inform selection of proxy product
  - Which methods did early adopters switch from?
  - Which method's current users are most interested?
- Look at how uptake of proxy product changed over time
  - Uptake of new methods tends to follow an S-curve with slow initial uptake, then a surge in growth, then a plateau
  - Do not expect a new product to quickly reach volumes similar to those of a long-established product

TOP RECOMMENDATIONS

- In the selection of approach:
  - Decide upon and communicate a clear purpose and intended use
  - Select your approach based on this purpose AND resource constraints

- When conducting the forecast or market size estimate:
  - Consider method switching and effects on broader method mix
  - Reality test the forecast results (e.g., based on analysis of capacity, historical consumption of other products, funding realities)
    - *Especially important when forecast feeds into supply plan*

- In the communication and/or use of results:
  - Clearly articulate assumptions
  - Acknowledge uncertainty
  - Refresh the forecast once you know if assumptions were correct

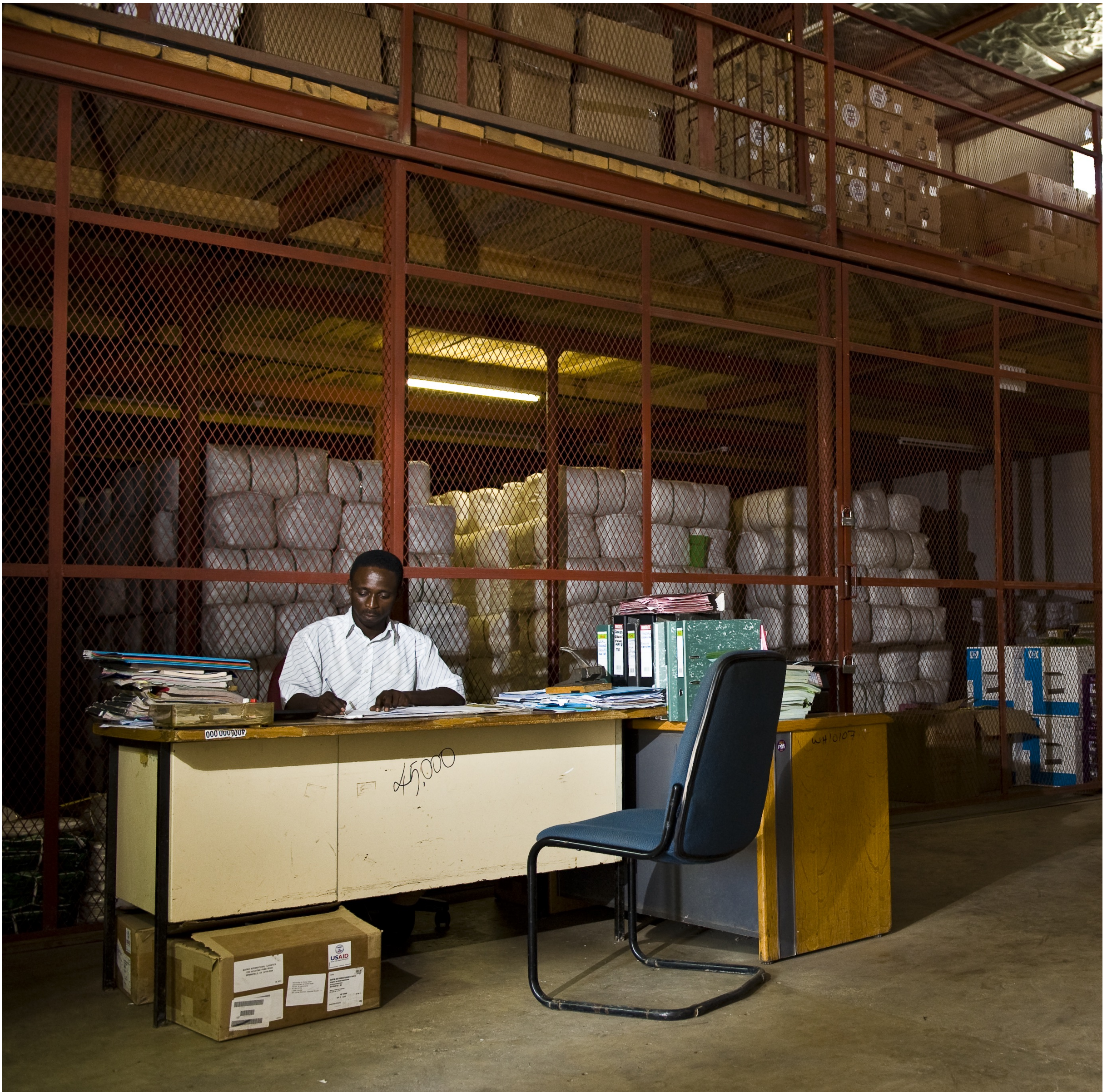

THANK YOU
